# Reduced Life Expectancy Model for Effects of Long Term Exposure on Lethal Toxicity with Fish

**DOI:** 10.1155/2013/230763

**Published:** 2013-12-26

**Authors:** Vibha Verma, Qiming J. Yu, Des W. Connell

**Affiliations:** ^1^Griffith School of Engineering, Griffith University, Nathan Campus, Brisbane, Queensland 4111, Australia; ^2^Griffith School of Environment, Griffith University, Nathan Campus, Brisbane, Queensland 4111, Australia

## Abstract

A model based on the concept of reduction in life expectancy (RLE model) as a result of long term exposure to toxicant has been developed which has normal life expectancy (NLT) as a fixed limiting point for a species. The model is based on the equation (LC_50_ = *a* ln(LT_50_) + *b*) where *a* and *b* are constants. It was evaluated by plotting ln LT_50_ against LC_50_ with data on organic toxicants obtained from the scientific literature. Linear relationships between LC_50_ and ln LT_50_ were obtained and a Calculated NLT was derived from the plots. The Calculated NLT obtained was in good agreement with the Reported NLT obtained from the literature. Estimation of toxicity at any exposure time and concentration is possible using the model. The use of NLT as a reference point is important since it provides a data point independent of the toxicity data set and limits the data to the range where toxicity occurs. This novel approach, which represents a departure from Haber's rule, can be used to estimate long term toxicity from limited available acute toxicity data for fish exposed to organic biocides.

## 1. Introduction

Toxicity is a function of both exposure time period and concentration or dose [1–4]. Nevertheless most of the toxicological data are based on the quantitative relationship between concentration or dose and adverse effect without consideration of the exposure time period [5–7]. Often imprecise terms such as acute, subacute, subchronic, and chronic are used to describe the exposure time [8]. It is not common to evaluate time as a quantifiable variable of toxicity and often the conditions of toxicity testing are not constant so time cannot be effectively quantified [9].

However there have been studies where exposure time has been evaluated as a quantifiable variable of toxicity [7, 10–12] and the relationship between exposure time and dose has been evaluated [6, 13–16]. But in these studies the exposure time is relatively short. While studies based on longer exposure time are important, particularly in the field of risk assessment with environmental contaminants where the exposure time is relatively long and the exposure level is often low. Information regarding the long term effects of exposure time with environmental chemicals is scarce [17].

The significance of exposure time in toxicological evaluations was first recognised by Warren [18] describing a relationship ((*C* − *C*_0_) × *t* = *k*) between exposure time (*t*) and exposure concentration as the lethal dose to 50% of organisms (*C*). In this equation *C*_0_ is a threshold concentration below which no apparent toxic effects are observed and *k* is a constant. But Haber [19] described it using the simplest form of the relationship between lethal concentration (or dose) and time as (*C* × *t* = *k*). This relationship was later described as Haber's rule of inhalation toxicology. Conventionally Haber's rule, or its modified forms, is used for empirical evaluation of the effect of exposure time on toxicity. To expand the relationship and its application, different variants have been proposed by various researchers [20–22].

However some researchers have noted that the product of (*C* × *t*) is not always constant [23] especially in the situation when exposure concentration is relatively low and/or exposure time is relatively long [24–26]. According to Haber's rule when the lethal concentration approximates zero, the exposure time must approximate infinity in order to maintain the product as a constant but this is not possible.

A reduced life expectancy (RLE) model has been proposed to study the effects of long term exposure [3, 27, 28]. According to this concept relatively long exposure times at low concentrations of toxicant cause reduction in the normal life expectancy (NLT) of the organism exposed. This model is based on a linear relationship of internal lethal concentration (ILC_50_) with the natural log of the exposure time with the normal life expectancy (NLT) as a limiting point. Unlike Haber's rule when the exposure concentration is zero, the exposure time is not infinite but the normal life expectancy of a particular organism.

The RLE model has been evaluated with zooplanktons using data from the scientific literature by plotting ln⁡ LT_50_ (exposure time for 50% lethality of the organisms) against LC_50_ (lethal concentration to 50% of organisms) in ambient water (Verma et al., 2012) [29]. The RLE model successfully fitted most of the zooplanktons data sets; however some sets of data were best fitted by a two-stage version of the RLE model [29]. The concept of reduction in life expectancy has also been used as a measure of toxicity by Mangas-Ramírez et al. [30] who studied the effect of cadmium on zooplankton. Gama-Flores et al. [31] observed a reduction in life expectancy due to exposure to cadmium with zooplankton.

The objective of this paper was to use life expectancy in the evaluation of the relationship between exposure time and exposure concentration utilising the fish toxicity data available in the scientific literature especially in the situation when exposure time is relatively long.

## 2. Theory

### 2.1. Reduced Life Expectancy Model

The RLE model proposed by Yu et al. [28] is based on a linear relationship between Internal Lethal Concentration (ILC_50_) and the natural logarithm of the corresponding exposure time (LT_50_). The ILC_50_ is preferred as it is the concentration of toxicant in the organism body at the target site [32–34]. Also it has the advantage that the kinetics effects of the uptake and bioconcentration processes have already been taken into account [3]. The relationship between ILC_50_ and LT_50_ for toxicants can be described by the following equation:

(1) $$\begin{array}{c} {\text{ILC}}_{50}=\frac{\left[\ln\left(\text{NLT}\right)-\ln\left({\text{LT}}_{50}\right)\right]}{d}, \end{array}$$

where ILC_50_ is the internal lethal concentration resulting in the death of 50% of the organisms exposed for the time LT_50_, LT_50_ is the exposure time, NLT is the time until 50% of the organisms die without exposure, and *d* is a constant. Constant *d* is a measure of toxicity and represents the reduction in life expectancy of the organism per unit concentration of the toxicant.

The value of LT_50_ represents a reduced life expectancy from the normal life expectancy (NLT) of the organism. When ILC_50_ is plotted against the LT_50_, the regression line can be extended up to the point where ILC_50_ becomes zero which corresponds to a toxicant free medium at which the organism would be expected to live its normal life expectancy. The model can be used to predict the reduction in life expectancy at different concentrations of toxicant in the environment. This model has already been tested using the data obtained from earlier work [35] and a high level of correlation between ILC_50_ and LT_50_ was observed [28].

However the limited availability of ILC_50_ data in the scientific literature [36] restricts the evaluation of the RLE model. Therefore the relationship has been extended from the ILC_50_ to the LC_50_ with the proposal that the relationship between LC_50_ and exposure time period (LT_50_) can be used to estimate the reduction in life expectancy of organisms [3].

The bioconcentration factor (*K*_*B*_) for aquatic organisms is the ratio between the concentration of toxicant in the organism (*C*_*B*_) and the concentration in water (*C*_*W*_) at equilibrium [3]. Thus

(2) $$\begin{array}{c} K_{B}=\frac{C_{B}}{C_{W}}, \end{array}$$

(3) $$\begin{array}{c} C_{W}\alpha C_{B}, \end{array}$$

where *C*_*W*_ is the lethal concentration (LC_50_) in water and *C*_*B*_ the corresponding Internal Lethal Concentration (ILC_50_). Thus,

(4) $$\begin{array}{c} {\text{LC}}_{50}\alpha {\text{ILC}}_{50}. \end{array}$$

The relationship obtained after replacing ILC_50_ with LC_50_ in (1) is given as follows:

(5) $$\begin{array}{c} \text{L}{\text{C}}_{50}=\frac{\left[\ln\left(\text{NLT}\right)-\ln\left(\text{L}{\text{T}}_{50}\right)\right]}{d}, \end{array}$$

or

(6) $$\begin{array}{c} \text{L}{\text{C}}_{50}=a\ln\left({\text{LT}}_{50}\right)+b, \end{array}$$

where *a* is −1/*d* and *b* is ln⁡(NLT)/*d*.

When LC_50_ is zero, the organism would have a normal life expectancy; thus

(7) $$\begin{array}{c} \text{L}{\text{T}}_{50}=\text{NLT}, \end{array}$$

(8) $$\begin{array}{c} \ln\left(\text{NLT}\right)=\frac{b}{a}. \end{array}$$

According to this model, at LC_50_ zero (toxicant free environment) organisms would be expected to live to their normal life expectancy. Therefore the model can be used to predict the reduction in the life expectancy at different concentrations of a toxicant in the external environment.

## 3. Methodology

### 3.1. Organisms and Toxicants Used for Evaluation

Fish were selected as study organisms since a large volume of toxicity data related to fish are available in the literature. The routes of toxicant uptake common to all fish are through gills, outer body surface, and food. The organic toxicants used in this study were those which had significantly different modes of toxic action and included various organic compounds including organophosphates, organochlorines, pyrethroids, and antiparasites.

### 3.2. Sources and Collection of Data

Toxicity data related to fish and organic toxicants were obtained from an extensive search of the literature. The data sets were used which included records of LC_50_ at various exposure durations. Most of the data sets had 4 points where the LC_50_ had been recorded at 24, 48, 72, and 96 hrs (Table 1) and only the 4 data sets on benzyl compounds with *Poecilia reticulata* had more than 4 points with exposure time longer than 96 hrs. The data sets in which LC_50_ required to cause toxicity did not change with exposure time were not processed. Various units for concentration were recorded such as mg/L, g/L, ppm, and ppb, so for consistency all units were converted into *μ*g/L. Similarly the exposure times were also expressed in various units (hours, minutes, and seconds), so all were converted into *day*. The Reported NLT data for each organism was also obtained from the literature. When ranges of NLT values were given, the average was calculated to obtain the Reported NLT. The temperature of ambient water used in the experiments ranged between 14°C and 30°C.

### 3.3. Processing of Data

The data sets available for each species of fish were used to evaluate the relationship between LC_50_ and ln(LT_50_) with the RLE Model as expressed in (6). The LC_50_ (*μ*g/L) was plotted against ln(LT_50_) and linear regression analysis was used to obtain the regression equation and correlation coefficient (*R* ^2^) using Excel. The regression line obtained was extended to the point where the LC_50_ became zero which corresponded to a toxicant free medium at which the organism would be expected to live its full NLT. The values of the slope (*a*) and intercept (*b*) were obtained from the regression equations. These values were then used to obtain the Calculated NLT of each species using (8). The characteristics resulting from this analysis are shown in Table 1. Only those data which had a minimum of four or more datasets available for each toxicant per fish species were considered to obtain the Calculated NLT (Table 1).

## 4. Results and Discussion

### 4.1. Model Evaluation

#### 4.1.1. Relationship between Exposure Time and Toxicity

**Figure 1:**
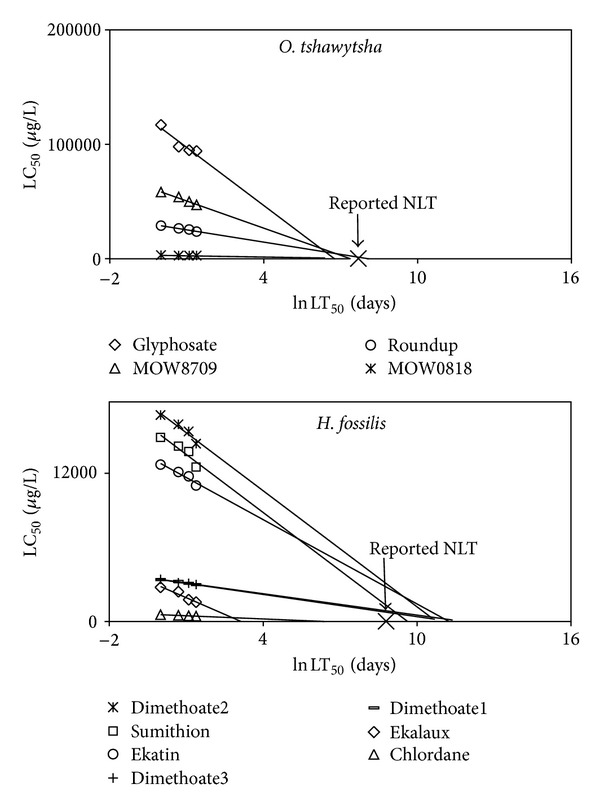
Examples of plots of LC_50_ against ln⁡ LT_50_ with the linear regression line and Reported NLT on the *x*-axis (Table 1).

The relationship of toxicity with exposure time was linear and had a negative slope in all cases; examples of the plots are shown in Figure 1. Characteristics of all plots of fish data are listed in Table 1. When *R* ^2^ was below 0.8, the relationship was not considered to follow a linear trend but only 3 datasets out of 67 were in this category. There are a variety of organic compounds (organophosphates, organochlorines, pyrethroids, and antiparasites) with different mechanisms of action but the relationship of LC_50_ and ln⁡ LT_50_ was linear with all toxicants. All plots irrespective of toxicant type had negative slopes indicating that lethal toxicity is related to exposure time and LC_50_ required to cause toxicity decreases consistently in a systematic pattern.

#### 4.1.2. The Use of the NLT as Reference Point and as a Limiting Point

According to Haber's rule (*C* × *t* = *k*) when the toxicant concentration is zero, the exposure time is infinity. But according to the RLE model, the NLT is a limiting reference point and when LC_50_ approximates zero, the exposure time (ln⁡ LT_50_) should be the organism's normal life expectancy (NLT). Thus maximum possible exposure time of any toxicant to any fish would be the NLT of that particular fish species. The use of NLT as a reference point is important since it limits the maximum exposure time from being infinity at zero exposure concentration (Haber's rule) to the NLT of the test organism (Figure 1).

### 4.2. Comparison of the Reported NLT with the Calculated NLT

**Figure 2:**
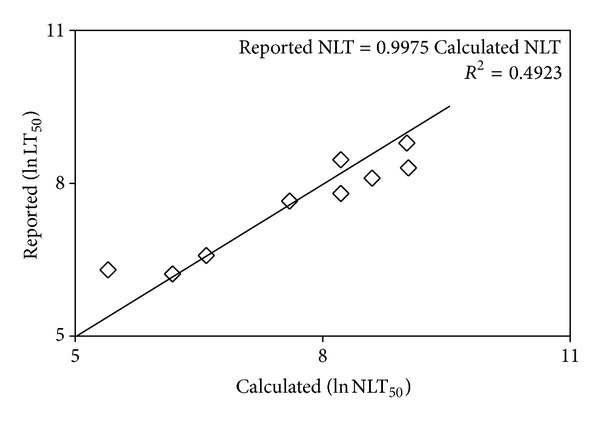
Plot of the Calculated NLT with the Reported NLT with linear regression line (Table 1).

The Calculated NLT for each fish species was obtained by the application of characteristics of the relationship obtained from regression analysis of data (Table 1) with (8). The Reported NLT ranging from 360 to 4700 days was plotted against the average Calculated NLT ranging from 120 to 8300 days for various fish species (Figure 2) giving a regression equation as follows:

(9) $$\begin{array}{c} \text{Reported}\;\;\text{NLT}=0.996\;\;\text{Calculated}\;\;\text{NLT},\;\;R^{2}=0.491. \end{array}$$

There are several sources of error in carrying out a comparison of the Reported NLT with that calculated from the RLE Model. An important factor is the decision on death during the toxicity test with fish considered dead becoming mobile when transferred to toxicant free water [67]. In addition there are many discrepancies regarding the Reported NLT. For example, Mugisha and Ddumba [37] reported the life expectancy of *O. niloticus* as 1 to 1.5 yrs, while Ofori-Danson and Kwarfo-Apegyah [68] have reported the life expectancy as 5 yrs. Changes in the temperature of ambient water affects fish life expectancy [69]. In warmer water the fish generally grows faster but the lifespan becomes shorter than in a cold environment [70]. Thus mortality can be related to growth and temperature [71]. It can be also expected that NLT would vary depending on such factors as oxygen availability, food, and so on. It is noteworthy that there are reports that the same fish species can have different life expectancies in different parts of the world [72–75].

Even though there are possible reasons for the Calculated NLT to differ from the Reported NLT as outlined above but overall the Reported NLT and Calculated NLT are in reasonably good agreement.

### 4.3. Application of the RLE Model

#### 4.3.1. Toxicity at Longer Exposures Times

**Figure 3:**
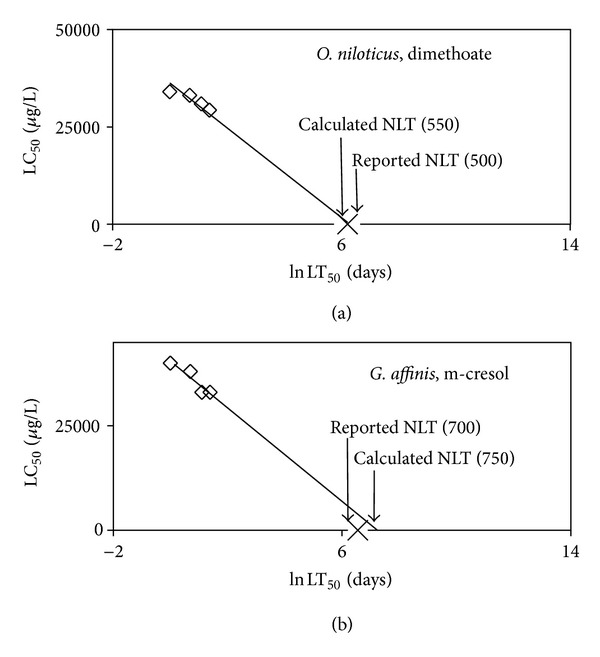
Example of plots of LC_50_ versus ln⁡ LT_50_ using the RLE model for estimation of long term toxicity.

The novel approach of the RLE model (6) allows the acute toxicity data available in the literature to be used for estimation of LC_50_ at other exposure times and also chronic toxicity. The equations obtained from regression analysis of LC_50_ versus ln⁡ LT_50_ (Table 1) can be used to estimate toxicity at any exposure time and exposure concentration for a particular fish species. For example plots of the LC_50_ versus ln⁡ LT_50_ using acute toxicity data of the toxicants dimethoate and m-cresol with the fish species *O. niloticus* and *G. affinis* are shown in Figures 3(a) and 3(b), respectively. It should be noted that the regression line intersects with the *x*-axis close to the Reported NLT (Figure 3) in both species. The regression equations obtained from this analysis are as described as follows:

(10) $$\begin{array}{c} O.\;\;niloticus\;\text{L}{\text{C}}_{50}=-5800\;\ln\;\text{L}{\text{T}}_{50}+36000\;R²=0.971, \end{array}$$

(11) $$\begin{array}{c} G.\;\;\;affinis\;\text{L}{\text{C}}_{50}=-1600\;\ln\;\text{L}{\text{T}}_{50}+15000\;R²=0.894. \end{array}$$

Using (10), where the slope (*a*) is −5800 and the intercept (*b*) is 36000, the LC_50_ of dimethoate with fish *O. niloticus* at any exposure time (ln⁡ LT_50_) can be calculated. Similarly using (11), the toxicity, as LC_50_ of m-cresol to *G. affinis* at any exposure time (ln⁡ LT_50_) can be obtained.

After plotting the toxicity data (LC_50_ versus lnLT_50_) obtained from the literature for a particular toxicant and fish species, application of (6) will give an estimate of toxicity of that particular toxicant for any exposure time particularly long ones.

When the toxicity information available is in the form of only one endpoint, most commonly the 96 hrs LC_50_, an estimation of toxicity at other exposure times is possible. This is done by using NLT of a particular organism as a limiting reference point. Firstly the LC_50_ versus ln⁡ LT_50_ can be plotted by using the 96 hr endpoint data from the literature, as one data point and the Reported NLT of the particular fish species as the second data point and an equation can be obtained from analysis of this relationship. This approach can be used to estimate toxicity at any exposure time, including long times for a particular fish species.

#### 4.3.2. Estimation of Normal Life Expectancy (NLT)

This approach can also be used to estimate the approximate NLT of a fish species. For the estimation of NLT, the first step is to plot LC_50_ versus ln⁡ LT_50_ recorded at various exposure times of as many data sets as possible related to that particular fish species. After calculation of the ln⁡ NLT_50_ by the use of (8) then the average of these individual NLT can be obtained, which is the average Calculated NLT for that particular fish species.

## 5. Conclusions

The relationship of toxicity with exposure time for fish using the equation given below was linear in almost all cases, had a negative slope, and is expressed as

(12) $$\begin{array}{c} \text{L}{\text{C}}_{50}=a\ln\left(\text{L}{\text{T}}_{50}\right)+b. \end{array}$$

This equation with parameters derived empirically for a particular fish species can be used to estimate toxicity at any exposure time particularly long exposure times.

The use of NLT as a limiting reference point is innovative since it limits the data to the range where toxicity occurs. In addition it provides a reference point which is independent from the toxicity data set, when the toxicant concentration is zero the life span is the normal life expectancy of the fish species. The equation above then becomes ln⁡ NLT = *b*/*a*. This is in contrast to Haber's rule from which an exposure time of infinity is obtained when the exposure concentration is zero.

In support of the relationship above the Calculated NLT and Reported NLT were in good agreement as expressed by the following relationship:

(13) $$\begin{array}{c} \text{Reported}\;\;\text{NLT}=0.998\;\;\text{Calculated}\;\;\text{NLT},\;R^{2}=0.4923. \end{array}$$

Available acute toxicity data can be used to calculate toxicity for a particular fish species exposed to organic biocides at any exposure time. Even with limited information (endpoint only) available the lethal toxicity estimation at any exposure is possible.

## Figures and Tables

**Table 1:** Regression analysis of the relationship between lnLT_50_ and LT_50_.

Fish species: organic compounds	Reported NLT *day * (lnNLT)	Calculated NLT *day *(lnNLT)**	Intercept (*b*)	Slope (*a*)*	Regression coefficient (*R* ^2^)	Reference
*Oreochromis niloticus *						
Dimethoate	500 (6.22) (Mugisha and Ddumba, 2006 [37])	480 (6.18)	36,000	−7,800	0.949	Phommakone, 2004 [38]
Dimethoate			34,000	−3,400	0.900	
Captan			580	−130	0.984	Boran et al., 2012 [39]
2,3,4,5-Tetrachlorophenol			310	−58	0.627	Holcombe et al., 1984 [40]

*Oncorhynchus gorbuscha *						
Glyphosate	600 (6.39) (Healey, 1986 [41]; Scott and Crossman, 1973 [42])	120 (4.78)	130,000	−50,000	0.962	Wan et al., 1989 [43]
MOW0818			3,300	−640	0.985	
MOW8709			60,000	−21,000	0.943	
Roundup			28,000	−3,000	0.957	

*Salmo gairdneri *						
2,3,4,5-Tetrachlorophenol	3300 (8.1) (Gorbach, 1961 [44])	5400 (8.6)	100,000	−9,400	0.832	Holcombe et al., 1984 [40]
Glyphosate			2,600	−360	0.977	Wan et al., 1989 [43]
MW0818			45,000	−10,000	0.986	
MOW8709			22,000	−1,800	0.982	
Roundup						

*Oncorhynchus tshawytesha *						
Glyphosate	2100 (7.65) (Moyle, 2002 [45]; Healey [41])	2000 (7.60)	110,000	−17,000	0.885	Wan et al., 1989 [43]
MW0818			3,000	−360	0.905	
MOW8709			58,000	−7,900	0.984	
Roundup			29,000	−3,600	0.978	

*Oncorhynchus keta *						
Glyphosate	2700 (7.8) (Scott and Crossman, 1973 [42])	3800 (8.23)	89,000	−14,000	0.998	Wan et al., 1989 [43]
MOW0818			2,400	−190	0.818	
MOW8709			44,000	−4,900	0.956	
Roundup			23,000	−4,400	0.980	

*Oncorhynchus kisutch *						
Glyphosate	1800 (7.51) (Scott and Crossman, 1973 [42])	2600 (7.88)	12,000	−2,000	0.984	Wan et al., 1989 [43]
MOW0818			3,300	−310	0.820	
MOW8709			48,000	−5,200	0.823	
Roundup			34,000	−5,100	0.972	

*Anguilla anguilla *						
Methylparathion	4700 (8.46) (EC Fisheries, 2013 [46])	3700 (8.22)	54,00	−870	0.977	Ferrando et al., 1991 [47]
Methildathion			3,700	−1,700	0.967	
Chlorpyrifos			1,200	−550	0.905	
Trichlorfon			4,900	−1,200	0.930	
Fenitrothion			340	−130	0.817	
Endosulfan			52	−1.3	0.742	
Diazinon			160	−59	0.981	

*Heteropenteus (Saccobranchus) fossilis *						
Dimethoate	6600 (8.79) (Flower, 1925 [48]; Altman and Dittmer, 1962 [49])	8100 (9.02)	17,000	−1,600	0.930	
Dimethoate			3,400	−300	0.966	Pandey et al., 2009 [50]
Dimethoate			3,400	−290	0.982	
Chlordane			500	−86	0.992	Pandey et al., 2008 [51]
Ekatin			13,000	−1,100	0.929	Verma et al., 1978 [52]
Ekalaux			2,800	−900	0.943	
Sumithion			15,000	−1,600	0.871	

*Poecilia.reticulata *						
Methylbenzoate	550 (6.3) (Fish Base [53])	220 (5.4)				
Benzonitrile			59,000	−12,000	0.931	Verhaar et al., 1999 [54]
Benzaldehyde			240,000	−42,000	0.908	
Benzylalcohol			23,000	−6,200	0.830	
Cypermethrin			640,000	−210,000	0.911	
Cypermethrin			3,200	−670	0.978	Gautara and Gupta, 2008 [55]
Cypermethrin			2,200	−360	0.956	
Cypermethrin			1,900	−280	0.991	
Cypermethrin			1,800	−210	0.962	
Cypermethrin			2,500	−520	0.978	
Cypermethrin			2,400	−460	0.983	

*Pimephales promelas *						
2-Allylphenol	720 (6.58) (Scott and Crossman 1973 [42])	730 (6.59)	32,000	−15,000	0.915	Holcombe et al., 1984 [40]
4-Tert-butylphenol			6,200	−800	0.989	
4-Chloro-3-methylphenol			14,000	−4,100	0.966	
4-Nitrophenol			65,000	−18,000	0.985	
2,3,4,5-Tetraphenyl			490	−40	0.882	
1,4-Dinitrobenzene			690	−60	0.982	

*Ictalutrus punctatus *						
1,4-Dinitrobenzene	2900 (8.30) (Jearld and Brown, 1971 [56])	N/A***	910	−170	0.971	Holcombe et al., 1984 [40]
2-Ethoxyethyllacetate			21,000	−4,200	0.946	

*Channa punctatus *						
Carbosulfan	2000 (7.60) (Nayak et al., 1999 [57])	N/A***	1,600	−1,000	0.830	Nwani et al., 2010 [58]
Glyphosate			41,000	−7,400	0.769	
Atrazine			63,000	−16,000	0.958	

*Cyprinus carpio *						
Dichlorvos	17160 (9.75) (Flower, 1925 [48])	N/A***	37,000	−1,100	0.932	Das, 2012 [59]
Dimethoate			1,900	−160	0.936	Singh et al., 2009 [60]

*Trichogaster trichopterus *						
Diazinon	1500 (7.31)	N/A***	40,000	−18,000	0.989	
Deltamethrin			300	−53	0.890	Hedayati et al., 2012 [61]

*Oncorhynchus mykiss *						
m-Cresol	1,300 (Scott and Crossman, 1973 [42])	N/A***	8,000	−3,000	0.999	Capkin et al., 2010 [62]
Deltamethrin			4.5	−3.4	0.982	Ural and Sağlam, 2005 [63]
Endosulfan			19	−13	0.987	Capkin et al., 2006 [64]

*Gambusia affinis *						
m-Cresol	700 (Krumholz, 1948 [65])	N/A***	40,000	−5,600	0.894	Sangli and Kanabur, 2000 [66]

*LC_50_ = *a* lnLT_50_ + *b*, **calculated from ln NLT_50_ = −*b*/*a*, (8), ***these species had <4 data sets.
